# The limits of chemosensation vary across dimensions

**DOI:** 10.1038/ncomms8468

**Published:** 2015-06-19

**Authors:** Brendan A. Bicknell, Peter Dayan, Geoffrey J. Goodhill

**Affiliations:** 1 Queensland Brain Institute, The University of Queensland, St Lucia, Queensland 4072, Australia; 2 School of Mathematics and Physics, The University of Queensland, St Lucia, Queensland 4072, Australia; 3 Gatsby Computational Neuroscience Unit, University College London, London WC1N 3AR, UK

## Abstract

Many biological processes rely on the ability of cells to measure local ligand concentration. However, such measurements are constrained by noise arising from diffusion and the stochastic nature of receptor–ligand interactions. It is thus critical to understand how accurately, in principle, concentration measurements can be made. Previous theoretical work has mostly investigated this in 3D under the simplifying assumption of an unbounded domain of diffusion, but many biological problems involve 2D concentration measurement in bounded domains, for which diffusion behaves quite differently. Here we present a theory of the precision of chemosensation that covers bounded domains of any dimensionality. We find that the quality of chemosensation in lower dimensions is controlled by domain size, suggesting a general principle applicable to many biological systems. Applying the theory to biological problems in 2D shows that diffusion-limited signalling is an efficient mechanism on time scales consistent with behaviour.

## Abstract (web summary)

Theoretical studies on chemosensation often invoke a model of three dimensional unbounded diffusion, but many biological problems involve two-dimensional diffusion in a bounded domain. Here Bicknell *et al.* present a model for chemosensation that covers bounded domains of any dimension, and apply it to biological problems in two dimensions.

## Graphical abstract

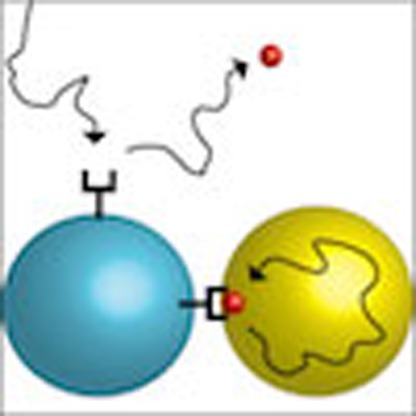

The ability of cells to sense their local chemical environment is fundamental to many biological processes. Chemosensation is impressively precise with, for instance, the flagellar motor in *Escherichia coli* reacting to changes in receptor occupancy of less than 1% (ref. 1), and *Dictyostelium discoideum* cells being capable of detecting gradients across the cell body corresponding to differences of only a few bound receptors^2^. Similarly, neuronal growth cones are exquisitely sensitive to soluble and membrane-bound guidance cues^3^,^4^ and lymphocytes are capable of accurate immunosurveilance by juxtacrine signalling^5^. However, at these limits of precision, biophysical considerations imply that there are significant differences in chemosensation as a function of dimension; this is important as biological sensing problems span one dimension (1D; for example, gene transcription^6^); two dimensions (2D; for example, membrane-bound reactions^7^; and receptor clustering^8^) and three dimensions (3D; for example, bacterial and eukaryotic chemotaxis^9^,^10^).

The quality of chemosensation is inherently limited by two different sources of variability: ‘reaction' noise associated with the stochastic nature of receptor binding, and ‘diffusion' noise arising from the motion of the ligands (here we do not consider noise from downstream signalling). In the biologically relevant regime, ligands diffuse slowly on the time scale of receptor activity. This implies that molecules that unbind from a cluster of receptors can rebind, producing temporal correlations in the statistics of receptor activation^11^. The nature of this noise is determined by the physics of diffusion, and so depends critically on both the dimension and spatial extent of the domain of diffusion (Fig. 1).

Berg and Purcell^11^ first derived a limit to concentration measurement in 3D using clever heuristic reasoning. Bialek and Setayeshgar provided a more rigorous answer which included the effects of reaction noise^12^, based on the fluctuation dissipation theorem (FDT)^13^ from statistical physics. Remarkably, to within a geometric factor, their result recovered the noise floor set by the ‘perfect instrument' considered by Berg and Purcell. Subsequent work extended these ideas to incorporate cooperative binding, gradient sensing and effects such as receptor diffusion and endocytosis^14^,^15^,^16^,^17^. Alternate probabilistic approaches^18^,^19^ corroborate the principal result of ref. 12, albeit including an extra factor in the limit of small numbers of receptors. However, although the 2D problem was touched on in early work^11^,^20^,^21^,^22^ and related subproblems appear in refs 17, 23, 24, 25, fuller extensions of these insights from 3D to other dimensions are lacking.

One example concerns two-stage capture models, which are based on the difference in diffusion as a function of dimension. Here the diffusing molecule first adsorbs to and diffuses on a lower dimensional surface before binding to a receptor. Since diffusion is a better search strategy in lower dimensions, this should decrease the mean time for the molecule to be captured and counted^20^. Paradoxically though, this coupling of 1D and 3D diffusion in gene transcription was found to be of little benefit to sensing due to a noise cost from increased temporal correlations in 1D^26^. Another example is a 2D result derived concurrently with our work. This explored the possibility that long time correlations may be avoided if ligands underwent endocytosis^27^. These authors made the claim that measurements of several hours may be required for accurate sensing in 2D.

These and other past studies have generally assumed an unbounded domain of diffusion, whereas the spatial extent of the intracellular space or membrane may in reality be limited. Additional length scales emerge in calculations of mean time to capture and diffusion-limited reaction rates in low dimensions^11^,^20^,^28^,^29^, although how this will affect temporal correlations from rebinding is unclear. This leaves open the question as to which principles will extend to a wide range of problems that occur in biological systems, such as a lymphocyte measuring the concentration of protein on the bounded 2D surface of a single cell.

Here we generalize the theory of ref. 12, using the FDT to investigate for the first time the physical limits to chemosensation in bounded domains of arbitrary dimensionality. We observe that the spatial information is encoded by the eigenfunctions of the Laplacian, explicitly revealing the dependence on the size of the domain in 1D, 2D and 3D. We derive general results for 2D sensing and demonstrate that there are two regimes of measurement time in low-dimensional systems. We verify our theoretical results numerically using particle simulations of Brownian dynamics in 1D and 2D, for single and multiple receptor systems. We show how the paradox due to 1D diffusion posed by ref. 26 can be resolved, and apply our 2D findings to the problems of cell recognition by natural killer cells in the immune system and axon guidance in retinotectal map formation. This demonstrates that under biological parameters diffusion-limited signalling can be an efficient mechanism for sensing in low dimensions.

## Results

### Derivation of theoretical results

We consider a cell attempting to measure the concentration *c* of a diffusible ligand by employing an array of *M* receptors. We assume that the average number of ligand molecules in the vicinity of the array is in excess of the number of receptors. Receptor states are denoted by binary variables *n*_*μ*_(*t*), equal to 1 when the receptor is bound and 0 when unbound. Individual receptors bind and unbind ligand with rate constants *k*_+_ and *k*_−_, respectively, so the equilibrium probability that an individual receptor is bound is given by 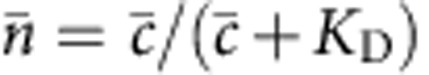, where *K*_D_=*k*_−_/*k*_+_ is the dissociation constant. The estimate of concentration is assumed to be based on an average of the total occupancy 
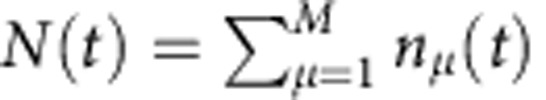
 over time *T*


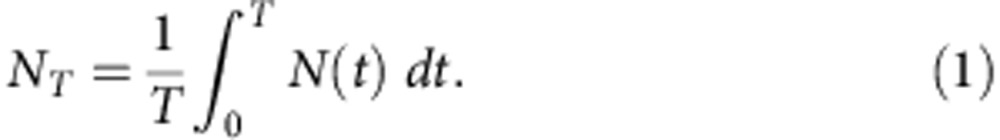


For long averaging times, the variance of *N*_*T*_ is given by 〈*δN*_*T*_^2^〉=2*τ*_*N*_〈*δN*^2^〉/*T* where 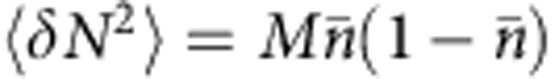 is the variance of *N* at equilibrium and *τ*_*N*_ is the correlation time defined by


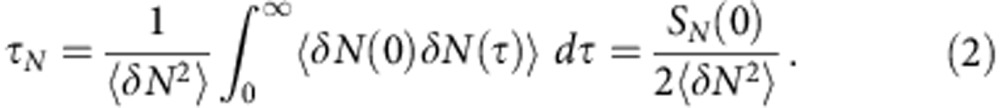


*S*_*N*_(*ω*) denotes the power spectrum of equilibrium fluctuations in occupancy 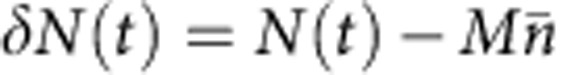, which by the Wiener–Khinchin theorem is the Fourier transform of the autocorrelation function. By inverting the expression relating 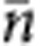 to 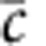, an estimate of the concentration can be formed as *c*(*N*_*T*_)=*K*_D_*N*_*T*_/(*M*−*N*_*T*_). To first order, the noise in the measurement *N*_*T*_ will propagate through to this estimate via the gain 
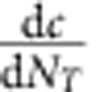
, giving rise to a fractional error


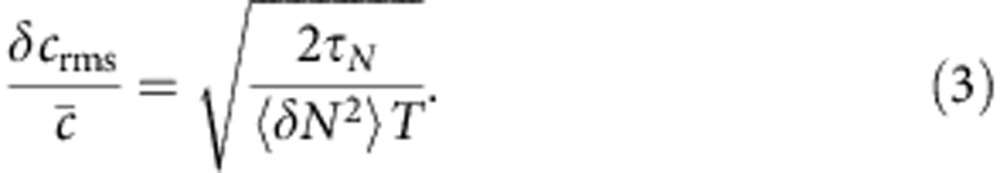


Equation (3) is valid as long as *T*≫τ_*N*_. We seek to determine *τ*_*N*_ as a function of the parameters of the model and the dimension and spatial extent of the domain of diffusion.

We use a mean field approach based on the framework of ref. 12, the accuracy of which increases with the number of receptors in the array. Using the FDT, we determine the power spectrum by finding the linear response of the occupancy to small perturbations in the free energy of binding *F*, expressed in the frequency domain by the susceptibility 
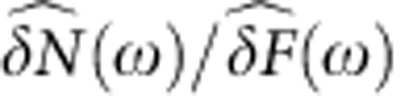
. This gives


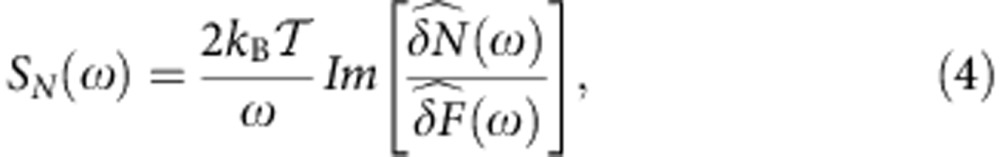


where 
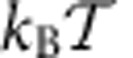
 is the thermal energy.

We distribute the receptors uniformly at radius *b* from the origin, up to a maximum number determined by the receptor radius *a* (assumed on the order of nanometres). The relaxation of the system to the equilibrium 
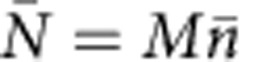
 is given by the kinetic equation


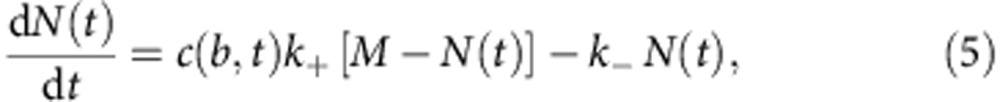


where *c*(*b*,*t*) denotes the average concentration of ligand at radius *b* from the origin. To capture the effect of diffusion on rebinding, activity of the receptor array feeds back into the binding rate by coupling with the diffusion equation. This is given in radial coordinates by


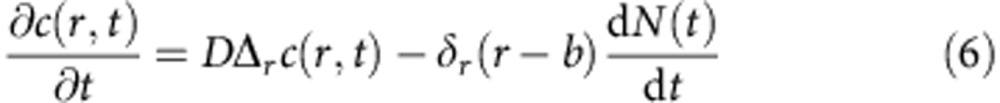


where 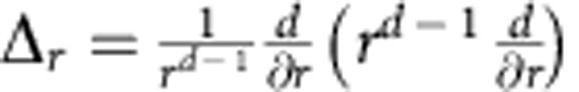 is the radial part of the Laplacian, and 
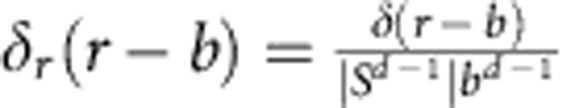
 is the radial delta function. The last term in equation (6) corresponds to the local change in concentration from binding and unbinding events. In this representation, step changes in *N*(*t*) from receptor activity result in impulses to the concentration corresponding to single molecules, which we average over the array. The resulting symmetry means the solution to this equation can be directly identified with the radial average that determines the binding rate in equation (5). In Supplementary Note 2, we use the 2D problem to illustrate that for large enough *M*, and *R*≫*a*, explicitly retaining the information in the model about the location and activity of individual receptors does not affect the results. Intuitively, this is because changes in the total state *N*(*t*) are comprised of many opposing binding/unbinding events distributed over the array, each contributing little in isolation. We also note that setting *M*=1 and replacing *b* with *a* gives a model for a single receptor at the origin similar to that in ref. 12. We make this interpretation in 1D, since in this case geometric constraints preclude the consideration of an array of many receptors.

Through detailed balance 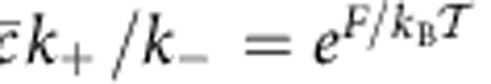, fluctuations in the rate constants are related to fluctuations in the binding energy via


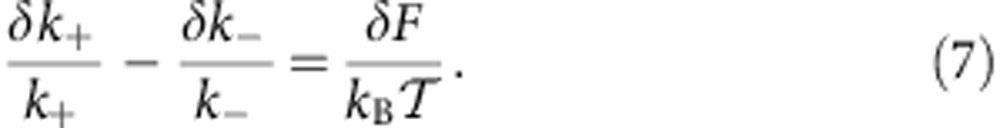


Thus, linearizing equation (5) at equilibrium gives a Langevin description of occupancy


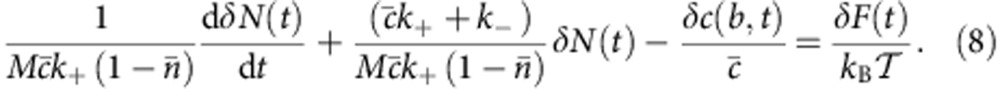


This small noise approximation is valid when there are many receptors, since then the fluctuations in occupancy will be small compared with the mean. The 1D result, with its single receptor, therefore comes with the caveat that less accuracy is expected in the approximation. We examine this numerically below.

Fourier transforming in time, the components are related in the frequency domain by


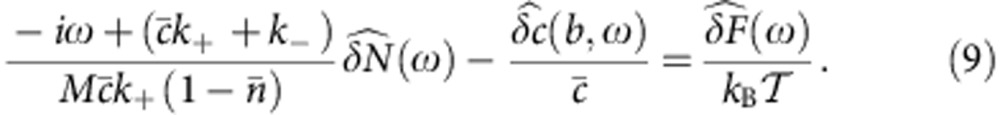


To determine the susceptibility and apply equation (4) requires an expression for concentration fluctuations in the frequency domain in terms of 
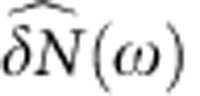
. It is here the dependence on the domain of diffusion becomes becomes explicit and we are forced to depart further from ref. 12. In infinite 3D space, this is handled in refs 12, 15, 16, 17 by Fourier transforming the diffusion equation in both space and time, leading to a self-energy term Σ(*ω*) arising from the (spatial) inversion integral. In 3D, in the relevant low frequency limit *ω*→0, corresponding to long averaging times, the resulting term quantifies the effect of diffusion as


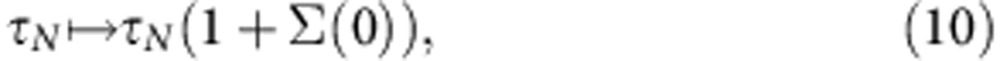


where *τ*_*N*_ is the correlation time in the fully reaction limited case (*D*→∞)^12^.

**Figure 1:**
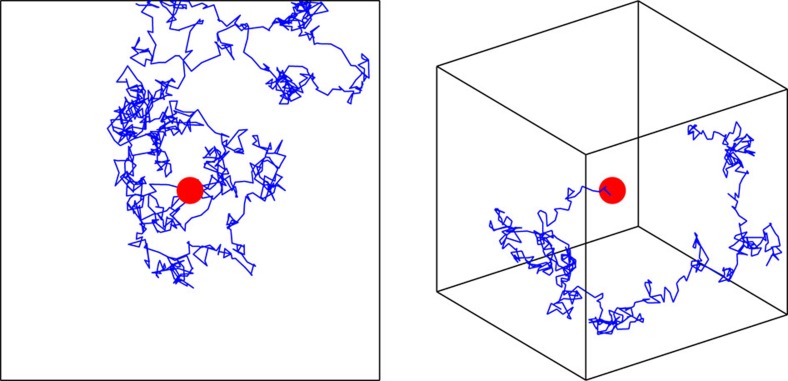
Noise due to diffusion is influenced by dimension and domain size. Representative trajectories (blue lines) of a molecule diffusing by Brownian motion about a single receptor (red circles) in bounded 2D and 3D domains. In 1D and 2D, return to the receptor is inevitable even in an unbounded domain, while in 3D the molecule quickly becomes lost in the bulk. The implication of these different regimes is that the ability of a cell to average over independent measurements is degraded in lower dimensions. Although intuitively it seems that the imposition of a boundary would amplify these effects, we find instead that for long averaging times the quality of chemosensation is improved. This is because the presence of a boundary reduces the relative contribution of recurrence to low frequency noise.

However, in 1D and 2D the self-energy diverges as *ω*→0, which corresponds to slow decay in the time domain. This implies that there are correlations that exist over extended averaging times, which degrade the ability of the cell to make independent measurements. At microscopic scales we can relate this to Polya's theorem for random walks on a *d*-dimensional lattice, which states that walks are recurrent for *d*≤2 and transient for *d*≥3 (see Fig. 1). The divergence is avoided when we impose a bounded domain on the system (which makes the recurrence happen in finite time). We take 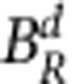, the *d*-dimensional ball of radius *R*, with an insulated boundary, as the domain of diffusion of ligand. This brings an additional spatial, and hence also temporal, scale to the problem. For a boundary at a distance *R* from a receptor or array to influence binding activity requires averaging times on the order of *T*>*R*^2^/2*dD*, determined by the characteristic time for diffusion in *d* dimensions. We proceed in this averaging time regime, although we return to this issue later. For shorter times, as far as a cell making a measurement is concerned, the system is indistinguishable from that of an unbounded domain.

We consider equation (6) for *r*∈[0,*R*] with the insulated boundary condition 
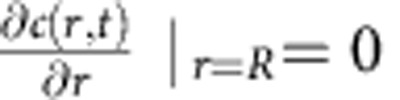
. Sturm–Liouville theory allows us to write the Green's function for this problem in terms of the eigenfunctions *φ*_*k*_ and eigenvalues 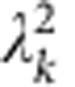 of the Laplacian Δ_*r*_. The Green's function is given by


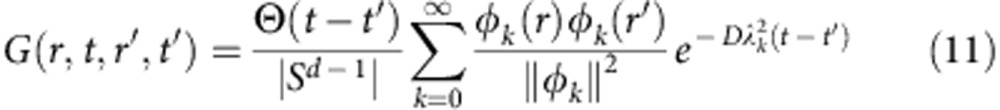


where Θ denotes the Heaviside step function and ||·|| is the *L*^2^ norm on [0,*R*] with respect to the weight function *w*(*r*)=*r*^*d*−1^. Thus, formally solving equation (6) through the Green's function and Fourier transforming in time we obtain


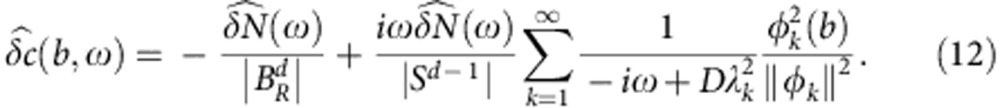


Substituting into equation (9) and applying the FDT, we arrive at


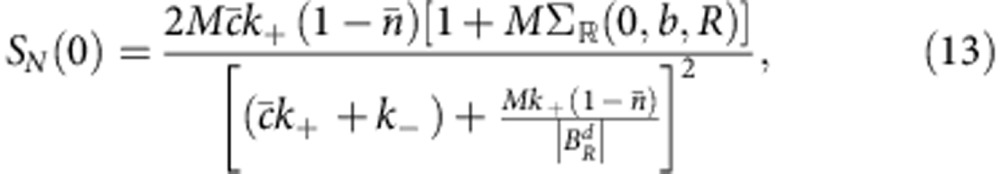


with the real part of the analogous self-energy term


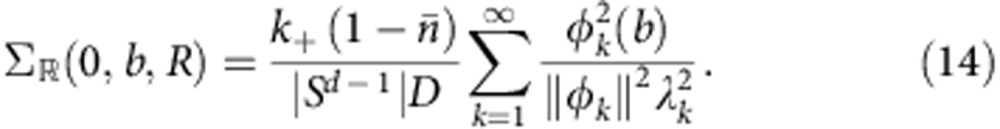


When there are sufficiently many ligand molecules that 
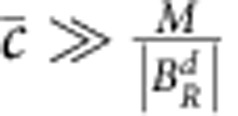
, we can neglect the second term in the denominator of equation (13). Doing so, and using the equilibrium condition 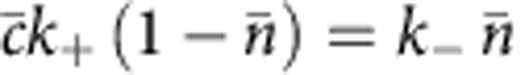, we arrive at a correlation time of


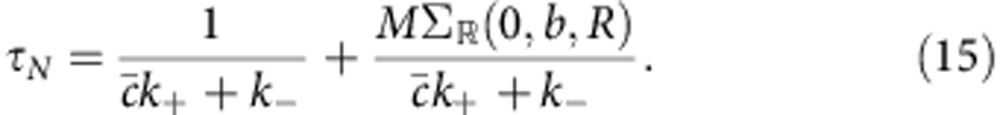


This is a more general form of equation (10), in which all of the dimensional and spatial dependence is encoded by the eigenfunctions specific to the domain.

In each dimension we must evaluate the sum in equation (14). The eigenfunctions are given on [0,*R*] by


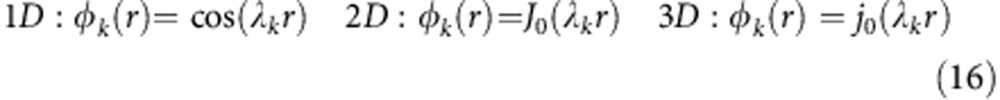


where *J*_0_ and *j*_0_ are the Bessel function and spherical Bessel function of the first kind of order 0. The eigenvalues are given by 
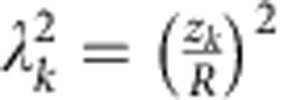
, where *z*_*k*_ are the positive zeros of *φ*′(*z*).

We derive closed forms for the sums in each case by manipulating them so they are given in terms of generalized Fourier series. Determining the functions represented by the Fourier series then allows us to reconstruct the original expressions (see Supplementary Note 1). This yields the solutions


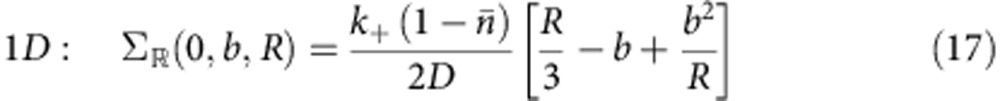



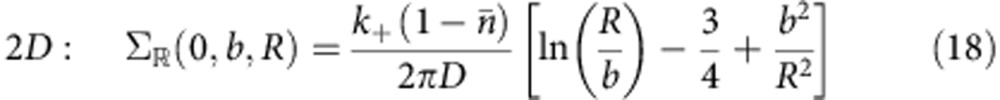



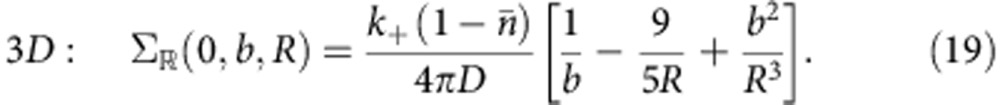


Then for *b*<<*R*, equations (3) and (15) give


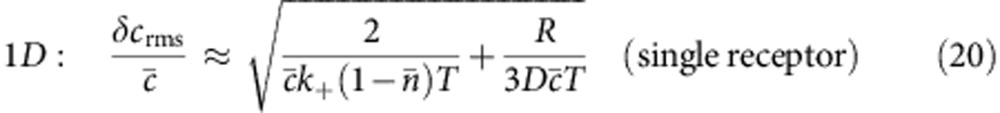



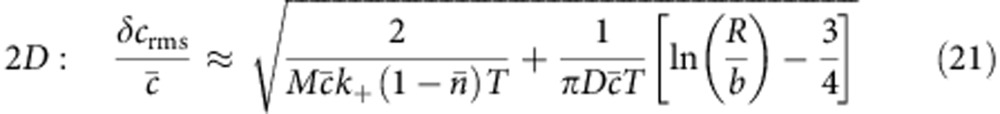



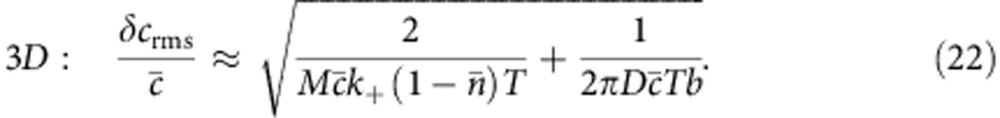


These expressions give the fractional errors in concentration measurement for long averaging times. The first term in each case is a consequence of the Markovian switching of the receptor. The influence of this can be reduced by increasing the number of receptors in the array, up to a maximum set by the radii of the receptors and the array. The second term is the unavoidable limit set by the physics of diffusion, and sets an upper bound on the precision of sensing. In all cases the error in the estimate decreases as 
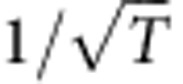
. In absolute terms, however, the influence of diffusion is controlled by the spatial properties of the system (as with the ‘tracking factors' of ref. 20 for mean time to capture). In 3D the size of the domain has little influence, and as *R*→∞ the noise floor set by the second term is exactly that found in ref. 18 for a sphere uniformly covered with receptors in infinite space. In lower dimensions, due to the qualitative difference in the statistics of diffusion, the measurement error is determined by the averaging time as well as the relevant biological scale.

Central to the arguments of Berg and Purcell is that concentration sensing is fundamentally limited by the diffusive arrival of molecules to the counting device. As shown in ref. 30, the fractional error for a cell that acts as a perfectly absorbing sink can be derived by considering the average current of molecules to its surface, 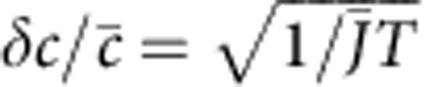. In 3D, for a spherical cell of radius *b<<R* the current is given by the Smoluchowski rate times the concentration, 
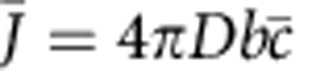
, so that 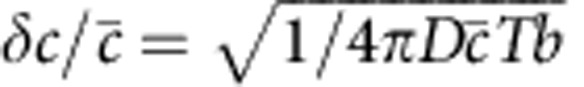. As a perfect absorber does not suffer counting noise in the form of rebinding this represents the absolute noise floor. Equation (22) shows that rebinding leads to a factor of 
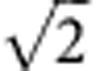
 increase in noise, or equivalently, an effective current 
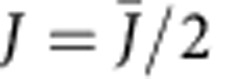
.

It turns out that exactly the same is true in 2D, except that the expression for 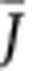 must be adjusted. There are several approaches for determining an analogous particle current in 2D, which differ by a small constant depending on the underlying geometry and approximations used (ref. 31, page 152). We follow ref. 11, who provided a calculation for the mean time to capture for particles diffusing in a disc with a circular absorber at the origin. In the limit that *R*≫*b* this is given (in our notation) as


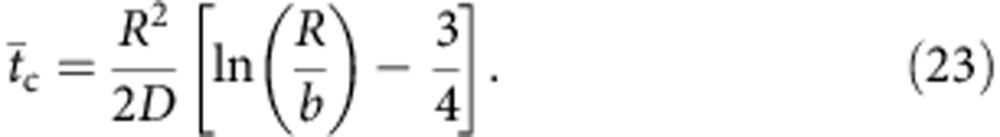


Thus we can approximate the average current to a circular array inside a disc by 
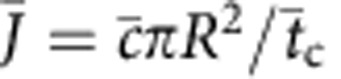
. In complete analogy with the 3D results, the expression 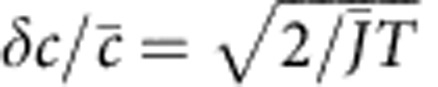 yields the second term in equation (21), providing a simple summary of the influence of rebinding for arrays in both 2D and 3D.

### Simulations

We performed particle simulations using Brownian dynamics in 1D and 2D to verify these results. In 2D, we simulated an ensemble of particles in a disc of radius *R* with a reflecting boundary. The diffusing particles interacted with 15 small receptor discs arranged in a uniform ring at the origin. We generated a long trajectory of the sum of receptor states and integrated the sample autocorrelation function to determine *τ*_*N*_. Parameters were chosen to be biologically relevant in the intermediate reaction/diffusion limited regime captured by the theory. We plot *S*_*N*_(0)=2*τ*_*N*_〈*δN*^2^〉 with the contribution from the reaction term plotted separately as a baseline.

**Figure 2:**
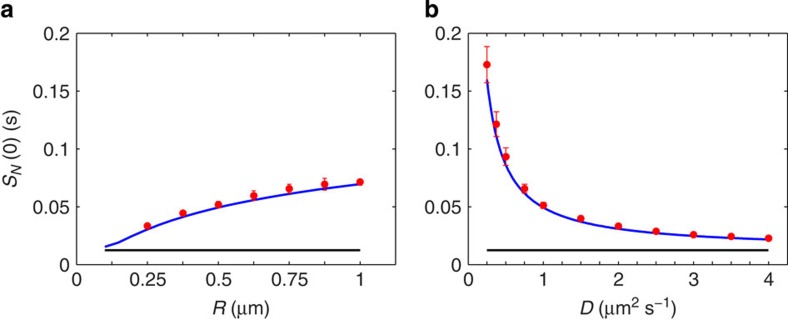
Simulations of a ring of 15 receptors in 2D. Predicted value of *S*_*N*_(0)=2*τ*_*N*_〈*δN*^2^〉 (blue lines) is plotted against simulations (red circles), demonstrating the effect of domain size (**a**) and the diffusion coefficient (**b**). The component of the prediction due to reaction noise alone is plotted as a baseline (black lines). Error bars are s.d. from *n*=10 simulations. Parameters: 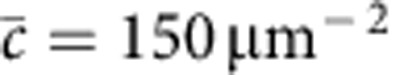, 
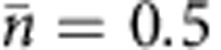
, *k*_+_=2 μm^2^ *s*^−1^, *k*_−_=300 s^−1^, *a*=10 nm, *b*=0.07 μm and *D*=1 μm^−2^ *s*^−1^ (**a**), *R*=0.5 μm (**b**).

The effect of diffusion on noise in the 2D system is shown in Fig. 2, both in terms of domain size and the diffusion coefficient. The logarithmic dependence on domain size is clear (Fig. 2a), and we find excellent agreement between theory and simulation even with this relatively small array. As predicted by the theory, the noise decreases rapidly with increasing speed of diffusion as molecules are cleared more quickly from receptors after unbinding (Fig. 2b). Reducing the ligand concentration, we find that the macroscopic treatment of diffusion in the theory yields accurate results down to 
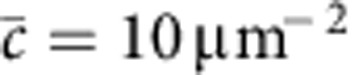
 and even lower concentrations (Supplementary Fig. 1).

**Figure 3:**
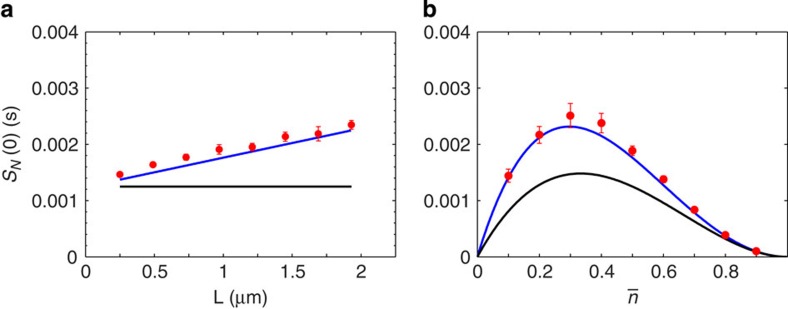
Single receptor system in 1D. Simulations (red circles) show a linear increase in noise with domain size (**a**). In the absence of spatial averaging over an array, the contribution from reaction noise (black lines) makes a larger contribution to the total (blue lines). The theory gives a good approximation over a range of mean occupancies (**b**). Error bars are s.d. from *n*=10 simulations. Parameters: 
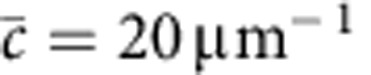
, *k*_+_=10 μm s^−1^, *k*_−_=200 s^−1^, *a*=5 nm, *D*=1 μm^−2^ s^−1^ and 
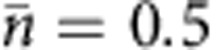
 (**a**), *L*=1 μm (**b**).

We noted earlier that the single receptor result in 1D was not expected to be as accurate as those for multiple arrays. In light of recent 3D results using alternate methods^18^,^19^, we anticipated that an extra dependence on mean occupancy may be lost in the linearisation (equation (8)). We simulated a single receptor on a line segment of length *L*, both to confirm the spatial dependence of the result and to test the closeness of the approximation. The predicted linear increase in noise with domain size is shown in Fig. 3a, showing close agreement under these parameters (
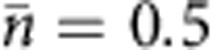
). We tested the dependence on mean occupancy by varying the concentration about 
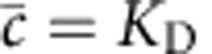
. Some discrepancy with the theory is seen, with an underestimation of the noise for 
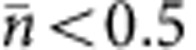
. However, the differences are small and do not affect the interpretation of our results.

We used the strong spatial dependence and computational simplicity of the 1D problem to probe the regime of averaging times *T*<*R*^2^/2*D*. In ref. 26 an alternate approach in 1D yielded an optimal estimate for sensing by a single receptor in an unbounded domain of


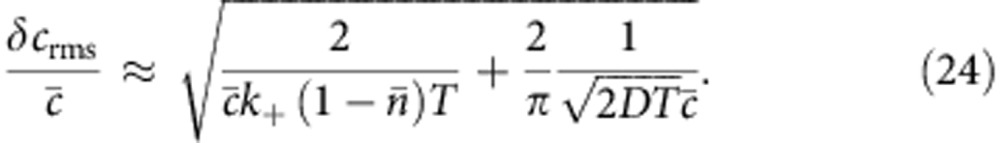


**Figure 4:**
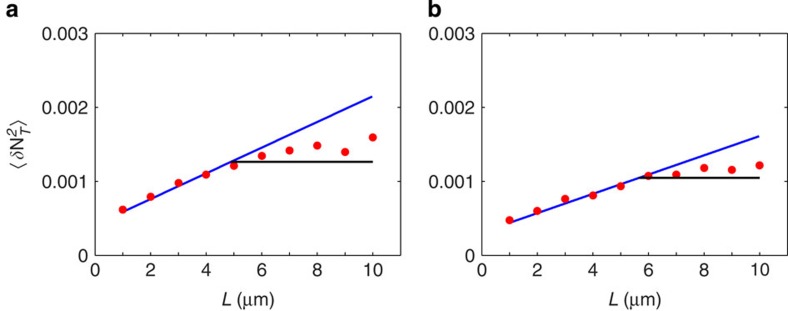
Two regimes of averaging time dependence in 1D. The measurement variance 
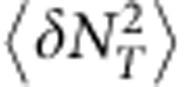
 is estimated from three thousand trajectories with fixed averaging times *T*=3 s (**a**) and *T*=4 s (**b**) (red circles). When *T*>*R*^2^/2*D* the boundary suppresses noise from diffusion, consistent with equation (20) (blue lines). For *T* < *R*^2^/2*D* the noise becomes independent of domain size and is bounded below by equation (20) with an effective domain size 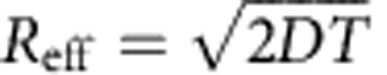 (black lines). Parameters: as in Fig. 3a.

In this case temporal correlations that arise from diffusion lead to a deleterious dependence on averaging time. We recover this expression almost exactly from equation (20) with an effective domain size 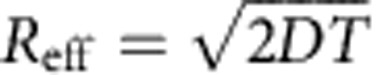, consistent with the splitting of averaging time regimes. To investigate this numerically we estimated the variance 
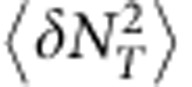
 by making the measurement given by equation (1) for 3,000 trajectories of fixed averaging times *T*=3s and *T*=4s (Fig. 4). When the domain is large, the variance becomes independent of *L*=2*R* and is bounded below by the optimal estimate for the unbounded domain. However, once the boundary becomes close enough to suppress the effects of recurrence at 
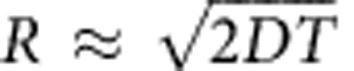
, the noise decreases linearly. Moving to 2D, substituting 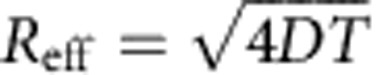 into equation (21) yields an averaging time dependence of the diffusion noise that is bounded between the 1D and 3D cases. Written in full this has the form


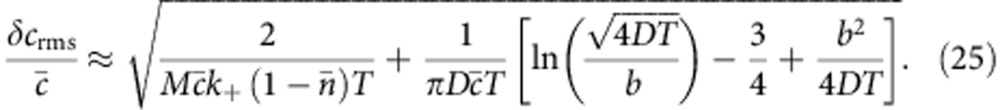


We predict that, as in 1D, this represents a lower bound for the noise in large domains or for shorter averaging times. We note that the logarithmic term in this expression is of a similar form to the result of ref. 27 in the case where the averaging time is much shorter then the characteristic time for endocytosis of ligand.

### Biological implications

The results above reveal the competing factors that contribute to the quality of chemosensation. The implications of this in 3D have been discussed previously, with evidence suggesting that bacterial systems, with averaging times on the order of seconds, may be operating close to the physical limit^11^,^12^. Our results show that in 3D the simplifying assumption of an unbounded domain is benign in most biological cases. We now consider some examples of low-dimensional sensing to draw out the dependencies that are most relevant in determining sensing precision under biological parameters. A key question is the extent to which noise limits persist in 2D, where processes such as juxtacrine signalling occur over minutes, but diffusion can be extremely slow. We consider three examples: DNA binding, natural killer cells in the immune system and axon guidance.

A 1D problem that has attracted notice^26^ is based on the observation that molecules slide along DNA. This type of two-stage capture has been proposed as a mechanism for increasing the efficiency of gene transcription, as it provides a larger effective target for transcription factors diffusing in the cytoplasm^6^. Treating the DNA molecule as infinitely long, the noise floor for measurement in 1D is given in ref. 26 by the second term in equation (24). The slow decrease in error with averaging time represents the penalty for averaging the signal for a time shorter than the correlation time, which, in this case, is infinite. 26 showed that this noise cost substantially reduces the benefit of two-stage capture. However, following the calculations in ref. 26 in the regime in which 1D diffusion dominates, using equation (20) we find that for an average sliding length *b* on a DNA segment of length *L*, the effective target size for the arrival of transcription factors from the bulk is given by 
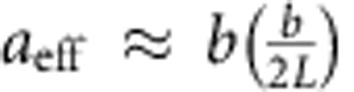
. That is, the size of the binding site is replaced by the sliding length scaled by the relative domain size. Because the target size strongly controls the noise in 3D, it is the relevant biological scale that determines the efficiency. For example, if transcription factors with sliding lengths *b*≈10–100 nm (ref. 26) are targeting an exposed promoter site in a nucleosome-depleted region of length *L*≈50–100nm (ref. 32) the noise reduction will be considerable, and greater still with mixing in the 3D bulk. This example demonstrates that all of the spatial properties of the system need careful consideration when moving to lower dimensions.

An example in which fast and reliable 2D sensing is a necessity is the inhibition of the cytotoxic response of natural killer cells in the immune system. Upon conjugation with a target cell, natural killer cell inhibitory receptors rapidly form microclusters, where it is suggested inhibitory signals from ligation of cognate major histocompatibility complex (MHC) class I protein are locally balanced against activating signals^33^,^34^. When MHC expression is below a threshold, activating signals lead to maturation of the synapse and lysis of the target cell. This serves as a defence against virally infected or cancerous cells that have down-regulated surface levels of MHC, whose role is to present antigens to T-cells. The canonical experimental system involves the binding of MHC protein HLA-C by inhibitory killer-cell immunoglobulin-like receptors (KIRs). Compared to similar 2D receptor–ligand systems, binding is very low affinity (minimal estimates *k*_−_≈2 s^−1^), suggesting that the concentration measurement is diffusion limited by design^35^. The diffusion coefficient of GFP-tagged HLA-C in the membrane of a human B-cell line is *D*≈0.3 μm^2^ s^−1^ (ref. 36). A sharp threshold between life and death of target cells interacting with natural killer cells has been observed *in vitro* at HLA-C concentrations on the order of 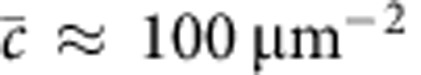 (refs 37, 38), demonstrating that natural killer cells are capable of reliably discriminating between cells that have a 10% difference in concentration. To estimate the noise floor, we use a disc of radius *R*=10 μm to represent the membrane of an (unwrapped) target cell, and *b*=0.05–0.5 μm the size of the microcluster that determines the flux of ligand across the receptors. Under these parameters, within the first two minutes of conjugation an optimal estimate could be formed with a fractional error 
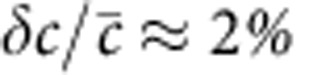
, which depends only weakly on the size of the cluster. The signal integration in this case may be provided by the level of activation of downstream components such as SHP-1 or Vav1. Although the specifics of decision making in natural killer cells are unknown, detailed simulations of the signalling network that employ the process of equilibrium binding we consider here have reproduced profiles of Vav1 activity consistent with experimental data and the threshold response to MHC^39^,^40^. The fast receptor–ligand kinetics and modest limitations placed by noise from diffusion make this a viable mechanism for efficient decision making in the early synapse. It will be interesting to compare this performance to that of other possible hypotheses as to the sensing mechanism.

In ref. 27, the authors give the example of axon guidance in retinotectal map formation to illustrate their 2D results. This is a paradigmatic problem of chemosensing, in which the tips of developing axons expressing Eph receptors navigate by sensing ephrin concentrations on the membranes of tectal cells over which they crawl. It is suggested in ref. 27 that noise from diffusion limits the growth rate of axons in this system. However, a rough estimate from equation (21) shows that this is unlikely, taken on its own. On reaching the tectum, axon growth slows to rates of 0.2–0.3 μm min^−1^ (refs 41, 42). If the limiting factor for an axon navigating over a bed of tectal cells each ∼10 μm in diameter was measurement noise, then the noise would need to be severe. For the spatial parameters, we take *R*=10 μm to be the effective radius of an unwrapped tectal cell, and *b*=0.1 μm for the radius of a small receptor array of the width of a filopodium. Ephrin-As are GPI-linked, so we expect relatively fast diffusion in the range *D*=0.1–1 μm^2^ s^−1^. For concentrations spanning 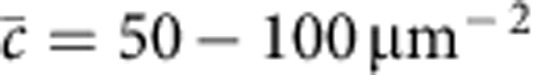, a measurement could in principle be made with a fractional error of 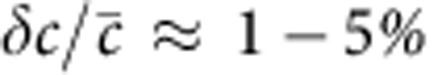 in only 2 min. While this is insufficient to explain axon growth rates, measurement on this time scale is consistent with the lifetime of individual filopodia as they probe the environment. A model of sensing that is more targeted towards the specifics of axon guidance will bring better understanding of the role of diffusion, and indeed the slow kinetics of Eph-ephrin binding, in this system.

These examples use the results we have derived to illustrate that, under biologically relevant parameters, accurate sensing can be achieved over measurement times consistent with behaviour. While a physical limit persists, a finite domain of diffusion suppresses the effects of recurrence so that the noise decreases as 
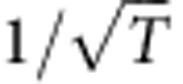
, just as it does in 3D. Although the diffusion coefficients of membrane proteins span several orders of magnitude, it is interesting to note that the ligands in the systems above are found at the high end of this range. HLA-C contains only a single transmembrane domain and Ephrin-As are GPI-linked, allowing for high mobility in both cases. Fast diffusion naturally reduces noise as in Fig. 2b, and confers the additional benefit of bringing the measurement into the 
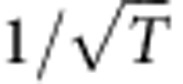
 regime of noise reduction much sooner. This makes the speed of diffusion the key parameter in the accuracy of 2D sensing, since unlike in 3D there is only a weak dependence on the size of the receptor array. An array in 2D need only be so large as to accommodate enough receptors to average away the reaction noise.

## Discussion

We used the fluctuation dissipation theorem to make explicit the spatial contribution to noise in chemosensing in terms of both dimensionality and domain size. Consistent with intuitions derived from Polya's theorem, we find a marked increase in temporal correlations in receptor activity in 1D and 2D systems compared with 3D; however, these effects are substantially mitigated when the domain is bounded. For a single receptor in 1D, the correlation time increases linearly with domain size, and the influence of the size of the target is minimal. By contrast, in 3D, the correlation time depends weakly on the domain size, and decreases with the size of the array. Wedged in between in 2D, the ‘critical dimension' for diffusion, we find these parameters in competition, though muted with a logarithmic dependence. The effect of target size can be understood in terms of the original arguments of refs 11, 20: in 3D a larger array provides a more effective sampling of the space. In lower dimensions, the size of the target is likely to be less important, as the statistics of diffusion should see molecules counted regardless. Counterintuitively, a boundary that forces molecules to remain near the receptors decreases the influence of temporal correlations, because the power of these correlations is pushed to higher frequencies which can be safely averaged away.

We make some simplifying assumptions in deriving these results. In writing equations (5) and (6) in terms of the average behaviour of the system, we have ignored the influence of any spatiotemporal correlations that may arise between receptors. However, the close agreement of the theoretical result with simulations of an array of discrete receptors in 2D suggests this is not a substantial factor. We have not explicitly considered the diffusion of receptors in the model. This simplified model allowed us to extract the essential features of noise limits across dimensions, and provides a useful point of reference for studying more dynamic features of receptor clustering in future work. For individual receptors diffusing within an array, or over the surface of an entire cell in 3D, we note that the governing equations still hold on average. If an array of receptors is itself moving with respect to the domain, such as the drift of a microcluster in the immune synapse, we would expect further noise reduction to a degree consistent with the increased flux of ligand. As long as motion of the cluster is slow relative to the motion of individual ligand molecules, the effect should be small. A fundamental assumption in this and most previous work is that a measurement is made by time averaging over receptor states. Analogous high-frequency filtering of the signal can be achieved, for example, by downstream processes that occur at finite rates. In 2D we have found that averaging times on the order of minutes are required, which raises the interesting question of what mechanisms might implement this operation. While beyond the scope of this work, if this line of inquiry imposes a constraint on signal averaging this would naturally increase the noise floor. The results of the 2D particle simulations agreed well with the theory over a physiological range of ligand concentrations spanning 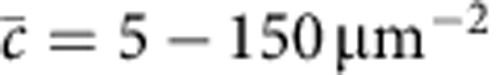. We do not consider here the limit of low concentrations in which the discrete nature of individual molecules becomes relevant. The methods employed by refs 18, 19, which are inherently more microscopic, may be better suited to this regime. Recent work using similar techniques in the technically demanding 2D case has provided relevant quantities such as isolated pair survival probabilities and reaction rate coefficients^24^,^25^. Extensions of this work in 2D will bring greater understanding to such problems as antigen sensing by T-cells, and we look forward to the insights gained from comparison with the results we have presented here.

The advantage of the thermodynamic method of ref. 12 that we have adopted here is the analytical freedom of working with a macroscopic description of receptor–ligand binding. We employed a simple and transparent numerical approach which shows that this gives a good representation of the system, particularly for multiple receptor arrays. Our treatment of concentration fluctuations as a boundary value problem brings further opportunities. For instance one can study the implications for gradient sensing directly by employing boundary conditions that give the desired steady-state concentration. Another interesting possibility is to study the noise in second messenger concentration in the intracellular 3D problem by using surface receptor activity to describe a fluctuating source on the boundary.

## Methods

### Simulations

We initialize an ensemble of non-interacting particles at a given concentration on a line segment of length *L* or disc of radius *R* centred at the origin. Initial positions are drawn from a uniform distribution. At each time step Δ*t*, the *i*th coordinate of the *j*th particle is updated by


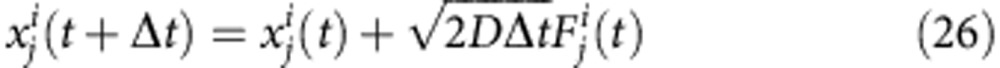


where *D* is the diffusion coefficient and 
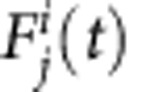
 is drawn from a normal distribution. In 1D if a particle crosses the boundary of the domain it is reflected back by


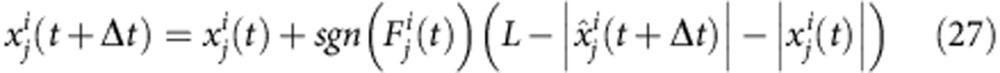


where 
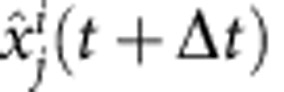
 is the coordinate of the point across the boundary. In 2D we reflect the particle about the inward pointing normal at the point of intersection of the line


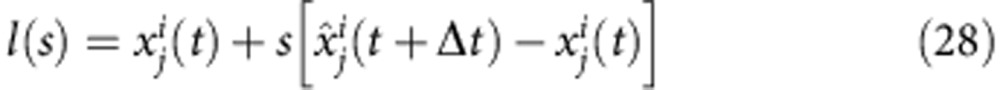


with the boundary of the disc.

Receptors with binary states *n*_*μ*_(*t*) are each represented by a capture radius *a* at the centre of the line segment in 1D or in a uniform ring of radius *b* in 2D. Each is initialized as *n*_*μ*_(*t*)=1 with probability 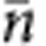, in which case a random particle is repositioned to the centre of the receptor. At each time step, if *n*_*μ*_(*t*)=0 a particle within radius *a* binds to the receptor with probability 
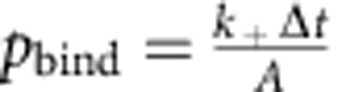
, where *A*=2*a* in 1D or *A*=*πa*^2^ in 2D. If binding is successful the particle is held at the centre of the receptor. If *n*_*μ*_(*t*)=1, the bound particle is released with probability *p*_unbind_=*k*_−_Δ*t*. The time step Δ*t* was chosen such that the probability of binding and unbinding events in a single time step was low over a range of parameters. This also ensured that a diffusing particle would take several timesteps to clear a receptor. These considerations led to Δ*t*=10^−5^ s in 1D and Δ*t*=5 × 10^−6^ s in 2D, which were used for all simulations. We confirmed that the results were robust to reducing the time step any further.

We generate a long trajectory of the sum *N*(*t*)=∑_*μ*_ *n*_*μ*_(*t*) over time *T* and then determine the correlation time *τ*_*N*_ by integrating the sample autocorrelation function of the trajectory. The simulation time was determined from preliminary data, where we estimated by eye the length of trajectory required for the evaluation of *τ*_*N*_ to converge. For most parameter values (Fig. 2b, Fig. 3b, Supplementary Fig. 1) *T*=100 s was sufficient. Capturing the effects of domain size required longer simulation time. Presumably this is because the effect of the increase in domain size is to add to the tail of the correlation function, which requires precise estimation to be picked up in the integral. As such, all data points in Fig. 2a and Fig. 3a of the main text were generated from simulation times *T*=200s, except those corresponding to *L*=1.75 μm and *L*=2 μm in Fig. 3a. These parameters required *T*=400 s and *T*=600 s, respectively. We confirmed that longer simulation times did not increase the estimate of *τ*_*N*_ for smaller values of *L*.

All simulations were performed using MATLAB (Mathworks). The simulation time required to generate a 100 s trajectory varied between 2 min in 1D with 1 receptor and 5 particles, to several hours in 2D with 15 receptors and 400 particles.

## Additional information

**How to cite this article**: Bicknell, B. A. *et al.* The limits of chemosensation vary across dimensions. *Nat. Commun.* 6:7468 doi: 10.1038/ncomms8468 (2015).

## Supplementary Material

Supplementary Information Supplementary Figures 1-5, Supplementary Notes 1-2 and Supplementary References.
