# Supplementary material for: The limits of chemosensation vary across dimensions
Source: Nat Commun. 2015 Jun 19;6:7468. doi: 10.1038/ncomms8468 (PMC4557358; doi:10.1038/ncomms8468)
Supplement: Supplementary Information — Supplementary Figures 1-5, Supplementary Notes 1-2 and Supplementary References. [file ncomms8468-s1.pdf]

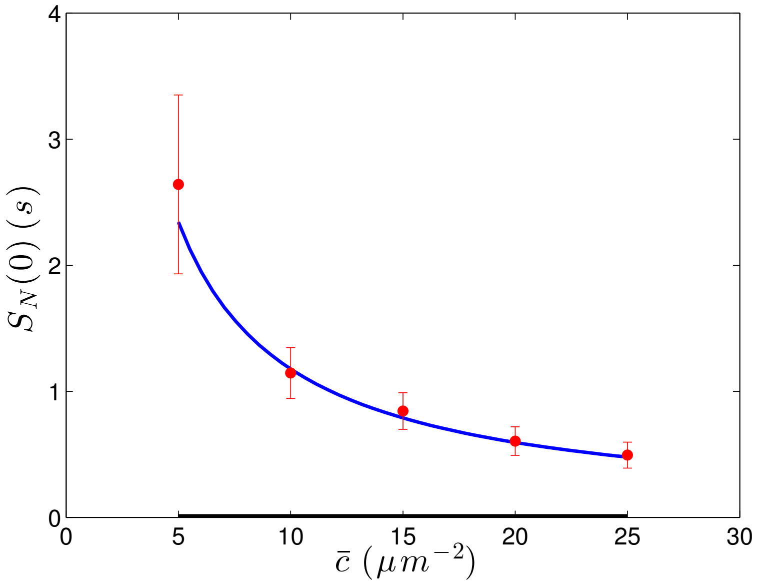

Supplementary Figure 1: **Simulations of a ring of 15 receptors in 2D at low ligand concentrations.** Predicted value of $S_N(0) = 2\tau_N \langle \delta N^2 \rangle$ (—) is plotted against simulations (•). The component of the prediction due to reaction noise alone is plotted separately (—). At these lower concentrations we used a larger simulation area so that the total number of molecules was sufficient to occupy the array. We have kept $\bar{n} = 0.5$ constant by varying the rate constant k_+. Error bars are SD from n=10 simulations. Parameters: $k_- = 300\,\mathrm{s}^{-1}$, $k_+ = k_-/\bar{c}\,\mu\mathrm{m}^2\,\mathrm{s}^{-1}$, $R = 2\,\mu\mathrm{m}$, $a = 10\,\mathrm{nm}$, $b = 0.07\,\mu\mathrm{m}$, $D = 1\,\mu\mathrm{m}^2\,\mathrm{s}^{-1}$.

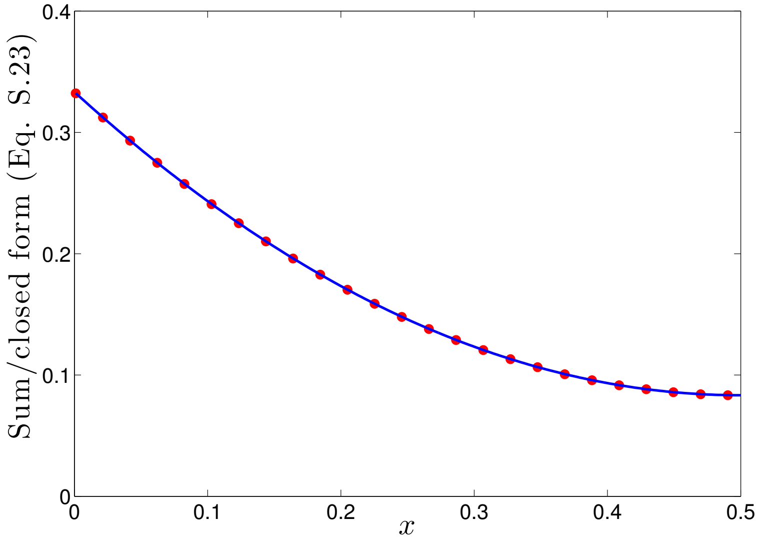

Supplementary Figure 2: **Numerical comparison of the sum (•) and closed form (—) in equation** (S.23)**.**

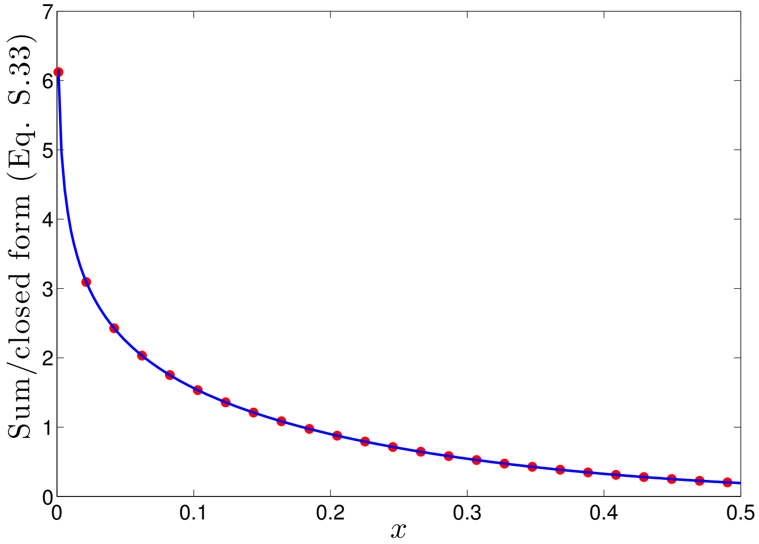

Supplementary Figure 3: **Numerical comparison of the sum (●) and closed form (—) in equation** (S.33)**, with** $g(x) = 1/2 - \ln(2)$**.**

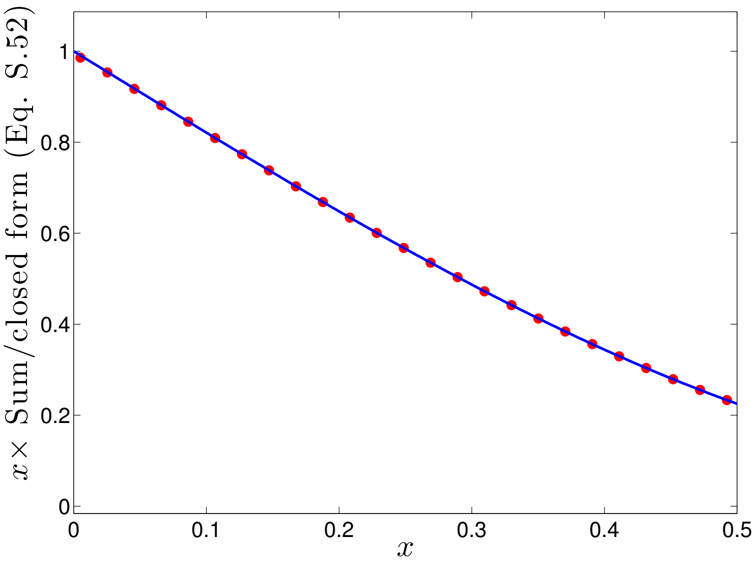

Supplementary Figure 4: **Numerical comparison of the sum (●) and closed form (—) in equation** (S.52)**.** For clarity we have multiplied through by x to remove the singularity.

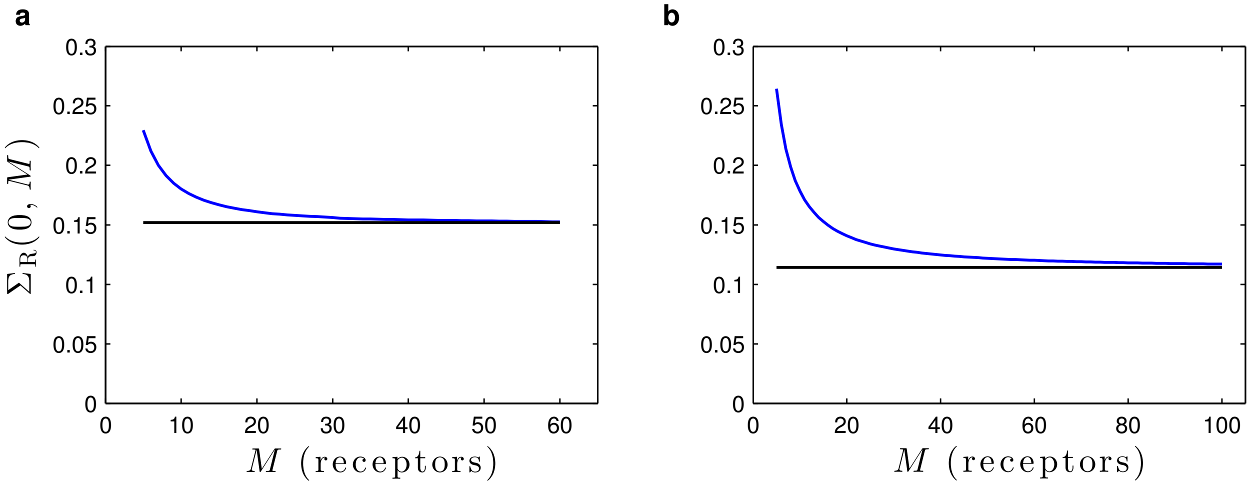

Supplementary Figure 5: **Saturation of noise reduction with receptor number in 2D**. Equation (S.70) is plotted as a function of M (—), when $b = 0.1\,\mu\mathrm{m}$ (**a**) and $b = 1\,\mu\mathrm{m}$ (**b**). The contribution from the first term alone is plotted separately (—). Parameters: $R = 10\,\mu\mathrm{m}$, $a = 5\,\mathrm{nm}$, $k_+ = 2\,\mu\mathrm{m}^2\mathrm{s}^{-1}$, $\bar{n} = 0.5$, $D = 1\,\mu\mathrm{m}^2\,\mathrm{s}^{-1}$.

Supplementary Note 1

The precision of chemosensation is limited by noise that arises from stochastic binding activity and the diffusion of ligand. We assume an estimation of concentration is formed by monitoring the total occupancy of receptors $N(t)$. By making a measurement over time T the cell can improve its estimate of concentration by averaging away the high frequency components of the noise. For long averaging times this leads to a first order fractional error given by equation (3) in the main text as

$$\frac{\delta c_{\mathrm{rms}}}{\bar{c}} = \sqrt{\frac{2\tau_N}{\langle \delta N^2 \rangle T}}. \tag{S.1}$$

The correlation time τ_N is a measure of the time taken for correlations in the state of the receptor array to decay away. Intuitively, the factor $\frac{2\tau_N}{T}$ in the fractional error is the inverse of the number of independent measurements that can be made in time T. The correlation time is determined by binding parameters, and is extended by the presence of diffusion which leads to rebinding of previously bound ligand.

Considering diffusion on B_R^d and M receptors arranged in a radially symmetric array of radius b, we describe the relaxation of the system by the coupled equations

$$\frac{dN(t)}{dt} = c(b,t)k_+[M - N(t)] - k_- N(t) \tag{S.2}$$

$$\frac{\partial c(r,t)}{\partial t} = D\Delta_r c(r,t) - \delta_r(r-b)\frac{dN(t)}{dt}. \tag{S.3}$$

Linearising these equations leads to equation (8) of the main text

$$\frac{1}{M\bar{c}k_+(1-\bar{n})}\frac{d\delta N(t)}{dt} + \frac{(\bar{c}k_+ + k_-)}{M\bar{c}k_+(1-\bar{n})}\delta N(t) - \frac{\delta c(b,t)}{\bar{c}} = \frac{\delta F(t)}{k_{\mathrm{B}}\mathcal{T}}, \tag{S.4}$$

whereby Fourier transforming in time relates the components in the frequency domain

$$\frac{-i\omega + (\bar{c}k_+ + k_-)}{M\bar{c}k_+(1-\bar{n})}\delta\hat{N}(\omega) - \frac{\hat{\delta c}(b,\omega)}{\bar{c}} = \frac{\delta\hat{F}(\omega)}{k_{\mathrm{B}}\mathcal{T}}. \tag{S.5}$$

Using the appropriate Green's function we solve for $\hat{\delta c}(b,\omega)$ explicitly as a function of receptor fluctuations, giving

$$\hat{\delta c}(b,\omega) = \frac{i\omega\Sigma(\omega,b,R)}{k_+(1-\bar{n})}\hat{\delta N}(\omega), \tag{S.6}$$

with

$$\Sigma(\omega,b,R) = \Sigma_{\mathbb{R}}(\omega,b,R) + i\Sigma_{\mathbb{I}}(\omega,b,R) \tag{S.7}$$

$$= \frac{k_+(1-\bar{n})}{|S^{d-1}|}\sum_{k=1}^{\infty}\frac{D\lambda_k^2}{\omega^2 + (D\lambda_k^2)^2}\frac{\phi_k^2(b)}{||\phi_k||^2} \tag{S.8}$$

$$+\,i\Big[\frac{k_+(1-\bar{n})}{\omega|B_R^d|}+\frac{k_+(1-\bar{n})}{|S^{d-1}|}\sum_{k=1}^{\infty}\frac{\omega}{\omega^2+(D\lambda_k^2)^2}\frac{\phi_k^2(b)}{||\phi_k||^2}\Big]. \tag{S.9}$$

Substituting into equation (S.5) and rearranging, the FDT gives

$$S_N(\omega)=\frac{2k_{\rm B}\mathcal{T}}{\omega}Im\Big[\frac{\delta\hat{N}(\omega)}{\delta\hat{F}(\omega)}\Big] \tag{S.10}$$

$$=\frac{2M\bar{c}k_+(1-\bar{n})[1+M\Sigma_{\mathbb{R}}(\omega,b,R)]}{[\bar{c}k_++k_-+\omega M\Sigma_{\mathbb{I}}(\omega,b,R)]^2+[\omega+\omega M\Sigma_{\mathbb{R}}(\omega,b,R)]^2} \tag{S.11}$$

Hence, using $\tau_N=\frac{S_N(0)}{2\langle\delta N^2\rangle}$, the correlation time is given by equation (15) in the main text,

$$\tau_N=\frac{1}{\bar{c}k_++k_-}+\frac{M\Sigma_{\mathbb{R}}(0,b,R)}{\bar{c}k_++k_-} \tag{S.12}$$

with

$$\Sigma_{\mathbb{R}}(0,b,R)=\frac{k_+(1-\bar{n})}{|S^{d-1}|D}\sum_{k=1}^{\infty}\frac{\phi_k^2(b)}{||\phi_k||^2\lambda_k^2}. \tag{S.13}$$

In this expression, the functions ϕ_k are the Neumann eigenfunctions of the radial Laplacian, and λ_k^2 are the positive eigenvalues. The norm $||\cdot||$ is the L^2 norm on $[0,R]$ with respect to the weight function $w(r)=r^{d-1}$.

Evaluation of sums

For fixed R, we regard equation (S.13) as a function of b. Rescaled, this is given in terms of the variable $x=\frac{b}{R}$ on $[0,1]$ by

$$\Sigma_{\mathbb{R}}(0,x)=\frac{k_+(1-\bar{n})R^{2-d}}{|S^{d-1}|D}\sum_{k=1}^{\infty}\frac{\phi_k^2(x)}{||\phi_k||^2 z_k^2}. \tag{S.14}$$

where the norm is over $x\in[0,1]$ without change in notation.

The eigenfunctions and norms are given on $[0,1]$ in each case by

$$1D:\quad \phi_k(x)=\cos(z_kx) \qquad ||(\cos)_k||^2=\frac{1}{2} \tag{S.15}$$

$$2D:\quad \phi_k(x)=J_0(z_kx) \qquad ||(J_0)_k||^2=\frac{1}{2}J_0(z_k)^2 \tag{S.16}$$

$$3D:\quad \phi_k(x)=j_0(z_kx) \qquad ||(j_0)_k||^2=\frac{1}{2(1+z_k^2)} \tag{S.17}$$

where J_0 and j_0 denote Bessel function and spherical Bessel function of the first kind of order 0, and z_k are the positive zeros of $\phi'(z)$. We derive closed forms for these sums by manipulating them so they are given in terms of generalised Fourier series. Determining the functions represented by the Fourier series then allows us to reconstruct the original expressions. We compare the solutions to numerical evaluations of the sums truncated at $k=2000$ terms, which was more than sufficient for visual detection of convergence.

1D

In this case

$$\sum_{k=1}^{\infty} \frac{\phi_k^2(x)}{||\phi_k||^2 z_k^2} = 2\sum_{k=1}^{\infty} \frac{\cos^2(k\pi x)}{(k\pi)^2} \tag{S.18}$$

$$= \Big[\sum_{k=1}^{\infty} \frac{1}{(k\pi)^2} + \sum_{k=1}^{\infty} \frac{\cos(2k\pi x)}{(k\pi)^2}\Big]. \tag{S.19}$$

For the second term, we consider

$$f(x) = \sum_{k=1}^{\infty} \frac{\cos(2k\pi x)}{(k\pi)^2} = 4\sum_{k=1}^{\infty} \frac{\cos(2k\pi x)}{(2k\pi)^2} \tag{S.20}$$

which we recognise as the Fourier series for the Bernoulli polynomial of order 2, so

$$f(x) = \frac{1}{6} - x + x^2. \tag{S.21}$$

The first term is given by

$$\sum_{k=1}^{\infty} \frac{1}{(k\pi)^2} = \frac{1}{6}, \tag{S.22}$$

so that as shown in Supplementary Fig. 2 we have

$$\sum_{k=1}^{\infty} \frac{\phi_k^2(x)}{||\phi_k||^2 z_k^2} = \frac{1}{3} - x + x^2. \tag{S.23}$$

Hence we find

$$\Sigma_{\mathbb{R}}(0, b, R) = \frac{k_+(1-\bar{n})}{2D}\Big[\frac{R}{3} - b + \frac{b^2}{R}\Big] \tag{S.24}$$

$$\tag{S.25}$$

which is equation (17) of the main text.

2D

Here we have

$$\sum_{k=1}^{\infty} \frac{\phi_k^2(x)}{||\phi_k||^2 z_k^2} = \sum_{k=1}^{\infty} \frac{J_0^2(z_k x)}{||(J_0)_k||^2 z_k^2}. \tag{S.26}$$

The sum diverges at $x = 0$, but for any interval $[\varepsilon, 1]$ with $\varepsilon > 0$, we can use the addition and recurrence formulae [2, 9.1.78, 9.1.27] to write

$$J_0^2(z_k x) = \frac{J_1(2z_k x)}{z_k x} - J_2(2z_k x) - 2\sum_{m=1}^{\infty} (-1)^m J_m^2(z_k x), \tag{S.27}$$

so that

$$\sum_{k=1}^{\infty} \frac{J_0^2(z_k x)}{||(J_0)_k||^2 z_k^2} = \frac{1}{x}\sum_{k=1}^{\infty} \frac{J_1(2z_k x)}{||(J_0)_k||^2 z_k^3} - \sum_{k=1}^{\infty} \frac{J_2(2z_k x) + 2\sum_{m=1}^{\infty}(-1)^m J_m^2(z_k x)}{||(J_0)_k||^2 z_k^2} \tag{S.28}$$

$$:= \frac{1}{x} f(2x) - g(x). \tag{S.29}$$

We consider the function

$$f(x) = \sum_{k=1}^{\infty} \frac{J_1(z_k x)}{||(J_0)_k||^2 z_k^3}. \tag{S.30}$$

Since $||(J_0)_k||^2 = ||(J_1)_k||^2$, this is a Fourier-Bessel series in J_1 with coefficients $c_k = \frac{1}{||(J_1)_k||^2 z_k^3}$. If f is represented by the series (S.30), then the orthogonality of the eigenfunctions implies that f satisfies the integral equation

$$\int_0^1 f(x) J_1(z_k x) x \, dx = \frac{1}{z_k^3}. \tag{S.31}$$

Carrying out the integrals, it can be shown that this has the solution

$$f(x) = -\frac{1}{2} x \ln(x) + \frac{1}{8} x^3 - \frac{1}{8} x, \tag{S.32}$$

to which the series (S.30) converges uniformly on $[0, 1]$. Then for $x \in [\varepsilon, \frac{1}{2}]$, equation (S.29) can be written

$$\sum_{k=1}^{\infty} \frac{J_0^2(z_k x)}{||(J_0)_k||^2 z_k^2} = -\ln(x) + x^2 - \ln(2) - \frac{1}{4} - g(x). \tag{S.33}$$

We were unable to derive a closed form for g, as defined by equation (S.28). However, numerical investigation of equation (S.33) and its derivative suggests this term is a small constant. Considering the limiting difference between the sum and the first group of terms in equation (S.33) as k becomes large, we find $g(x) \sim \frac{1}{2} - \ln(2)$. In fact, this holds numerically on $(0, 1]$ as a consequence of equation (S.30) holding on $[0, 2]$. It is this observation that suggested the form of the constant, since formally substituting $x = 1$ into equation (S.33) gives

$$g(x) = \frac{3}{4} - \ln(2) - 2\sum_{k=1}^{\infty} \frac{1}{z_k^2} \tag{S.34}$$

$$= \frac{1}{2} - \ln(2), \tag{S.35}$$

where we used the formula [3, 15.51] for the sum over zeros. The agreement between each side of equation (S.33) with this choice of g is shown in Supplementary Fig. 3.

Finally, we find

$$\Sigma_{\mathbb{R}}(0, b, R) = \frac{k_+(1-\bar{n})}{2\pi D}\left[\ln\left(\frac{R}{b}\right) - \frac{3}{4} + \frac{b^2}{R^2}\right] \tag{S.36}$$

$$\tag{S.37}$$

which is equation (18) of the main text.

3D

Here we have

$$\sum_{k=1}^{\infty} \frac{\phi_k^2(x)}{||\phi_k||^2 z_k^2} = \sum_{k=1}^{\infty} \frac{j_0^2(z_k x)}{||(j_0)_k||^2 z_k^2}. \tag{S.38}$$

Restricting again to an interval $[\varepsilon, 1]$, and using $j_0(z) = \frac{\sin(z)}{z}$ we can expand this as

$$\sum_{k=1}^{\infty} \frac{j_0^2(z_k x)}{||(j_0)_k||^2 z_k^2} = \frac{1}{x^2} \sum_{k=1}^{\infty} \frac{\sin^2(z_k x)}{||(j_0)_k||^2 z_k^4} \tag{S.39}$$

$$= \frac{1}{2x^2} \Big[\sum_{k=1}^{\infty} \frac{1}{||(j_0)_k||^2 z_k^4} - \sum_{k=1}^{\infty} \frac{\cos(2 z_k x)}{||(j_0)_k||^2 z_k^4} \Big] \tag{S.40}$$

$$= \frac{1}{2x^2} \Big[\sum_{k=1}^{\infty} \Big(\frac{2}{z_k^4} + \frac{2}{z_k^2} \Big) - f(2x) \Big], \tag{S.41}$$

where we define

$$f(x) = \sum_{k=1}^{\infty} \frac{\cos(z_k x)}{||(j_0)_k||^2 z_k^4}. \tag{S.42}$$

As above, since z_k are the zeros of $j_0'(z) = -j_1(z)$, and $||(j_0)_k||^2 = ||(j_1)_k||^2$, we try to express $f(x)$ in terms of a Fourier-Bessel series in j_1. Using the derivative formula for spherical Bessel functions [2, 10.1.23] (and uniform convergence to apply this term by term) we find

$$f(x) = \sum_{k=1}^{\infty} \frac{\cos(z_k x)}{||(j_0)_k||^2 z_k^4} \tag{S.43}$$

$$= \frac{d}{dx} \sum_{k=1}^{\infty} \frac{x j_0(z_k x)}{||(j_0)_k||^2 z_k^4} \tag{S.44}$$

$$= \frac{d}{dx} \Big[\frac{1}{x} \frac{d}{dx} \Big(x^2 \sum_{k=1}^{\infty} \frac{j_1(z_k x)}{||(j_1)_k||^2 z_k^5} \Big) \Big]. \tag{S.45}$$

We consider

$$g(x) = \sum_{k=1}^{\infty} \frac{j_1(z_k x)}{||(j_1)_k||^2 z_k^5}, \tag{S.46}$$

and as before we seek a function that satisfies the integral equation

$$\int_0^1 g(x) j_1(z_k x) x^2 \, dx = \frac{1}{z_k^5}. \tag{S.47}$$

Carrying out the integrals shows this has the solution

$$g(x) = -\frac{1}{280} x^5 + \frac{6}{100} x^3 - \frac{1}{8} x^2 + \frac{12}{175} x, \tag{S.48}$$

so that equation (S.45) gives

$$f(x) = -\frac{1}{8}x^4 + \frac{9}{10}x^2 - x + \frac{36}{175}. \tag{S.49}$$

For the first term in equation (S.41) we again use the formulae for sums over zeros [3, 15.51], which gives

$$2\sum_{k=1}^{\infty} \frac{1}{z_k^4} + \frac{1}{z_k^2} = \frac{1}{2^4(\frac{3}{2}+1)^2(\frac{3}{2}+2)} + \frac{1}{2^2(\frac{3}{2}+1)} \tag{S.50}$$

$$= \frac{36}{175}. \tag{S.51}$$

Finally,

$$\sum_{k=1}^{\infty} \frac{\phi_k^2(x)}{||\phi_k||^2 z_k^2} = \frac{1}{x} - \frac{9}{5} + x^2, \tag{S.52}$$

which we compare in Supplementary Fig. 4, where for clarity we have multiplied through by x to remove the singularity. Thus we find

$$\Sigma_{\mathbb{R}}(0, b, R) = \frac{k_+(1-\bar{n})}{4\pi D}\left[\frac{1}{b} - \frac{9}{5R} + \frac{b^2}{R^3}\right] \tag{S.53}$$

$$\tag{S.54}$$

which is equation (19) of the main text.

Supplementary Note 2

Derivation without spatial averaging

The radial symmetry arising from the spatial averaging in equations (S.2) and (S.3) allowed us to use a simple solution to the diffusion equation. Here we provide a derivation in which we explicitly consider the location of individual receptors and their influence on local concentration fluctuations. This shows that the averaging does not affect the results when M is sufficiently large. This setup mirrors that of Ref.[1] more closely, although we generalise to arbitrary dimension and domain size. We proceed in generality for a domain Ω, and then restrict our attention to the ring of receptors sensing in a disc that we considered in the main text.

We consider a cell attempting to measure the concentration c of a diffusible ligand in Ω by employing an array of M receptors located at points $\mathbf{x}_{\boldsymbol{\mu}}$. As before, the estimate of concentration is assumed to be based on an average of the total occupancy $N(t) = \sum_{\mu=1}^{M} n_\mu(t)$ over time T.

The evolution of the probability that an individual receptor is bound, given an initial state, is given by the kinetic equation

$$\frac{dn_\mu(t)}{dt} = c(\mathbf{x}_{\boldsymbol{\mu}}, t)k_+[1 - n_\mu(t)] - k_- n_\mu(t), \tag{S.55}$$

where $c(\mathbf{x}_{\boldsymbol{\mu}}, t)$ is the local concentration. Summing over all of the receptors, the relaxation of the system is given by

$$\frac{dN(t)}{dt} = \sum_{\mu} c(\mathbf{x}_{\boldsymbol{\mu}}, t)k_+[1 - n_\mu(t)] - k_- \sum_{\mu} n_\mu(t). \tag{S.56}$$

This is coupled to diffusion via

$$\begin{cases} \dfrac{\partial c(\mathbf{x}, t)}{\partial t} = D\Delta c(\mathbf{x}, t) - \displaystyle\sum_{\mu} \delta(\mathbf{x} - \mathbf{x}_{\boldsymbol{\mu}}) \dfrac{dn_\mu(t)}{dt} & \text{on } \Omega \\ \nabla c(\mathbf{x}, t) \cdot \mathbf{n} = 0 & \text{on } \partial\Omega, \end{cases} \tag{S.57}$$

where now a binding event results in an impulse to the concentration at the site of the receptor, and we do not average this over the array.

Linearising these equations and introducing fluctuations in the binding energy yields a Langevin description analogous to equation (S.4),

$$\frac{1}{M\bar{c}k_+(1-\bar{n})} \frac{d\delta N(t)}{dt} + \frac{(\bar{c}k_+ + k_-)}{M\bar{c}k_+(1-\bar{n})} \delta N(t) - \frac{1}{M} \sum_{\mu} \frac{\delta c(\mathbf{x}_{\boldsymbol{\mu}}, t)}{\bar{c}} = \frac{\delta F(t)}{k_{\mathrm{B}}\mathcal{T}}, \tag{S.58}$$

with Fourier transform

$$\frac{-i\omega + (\bar{c}k_+ + k_-)}{M\bar{c}k_+(1-\bar{n})} \delta\hat{N}(\omega) - \frac{1}{M} \sum_{\mu} \frac{\hat{\delta c}(\mathbf{x}_{\boldsymbol{\mu}}, \omega)}{\bar{c}} = \frac{\delta\hat{F}(\omega)}{k_{\mathrm{B}}\mathcal{T}}. \tag{S.59}$$

Notice that the only difference between equation (S.58) and equation (S.4) above is the term involving concentration fluctuations. However, this is just a discrete average over the array, and approaches the continuous average above as M becomes large. Once again we solve for this term by constructing a Green's function from the Neumann eigenfunctions ϕ_α of the Laplacian on Ω. The Green's function is given by

$$G(\mathbf{x}, t, \mathbf{x}', t') = \Theta(t - t') \sum_{\alpha} \frac{\phi_\alpha(\mathbf{x})\phi_\alpha(\mathbf{x}')}{||\phi_\alpha||^2} e^{-D\lambda_\alpha^2(t-t')} \tag{S.60}$$

where Θ denotes the Heaviside step function and $||\cdot||$ is the L^2 norm on Ω. α is a multi-index, dependent on the dimension, that runs over all eigenfunctions. Thus, formally solving equation (S.57), summing over the receptors and Fourier transforming in time we obtain

$$\sum_{\mu} \hat{\delta c}(\mathbf{x}_{\boldsymbol{\mu}}, \omega) = -\frac{M\delta\hat{N}(\omega)}{|\Omega|} + \sum_{\nu} i\omega\hat{\delta n}_\nu(\omega) \sum_{\mu} \sum_{|\alpha|>0} \frac{\phi_\alpha(\mathbf{x}_{\boldsymbol{\mu}})\phi_\alpha(\mathbf{x}_{\boldsymbol{\nu}})}{||\phi_\alpha||^2} \frac{1}{-i\omega + D\lambda_\alpha^2}. \tag{S.61}$$

We ensure convergence of the inner sum by truncating at the index Λ such that $\lambda_\alpha \leq \frac{\pi}{a}$ for all α. This is analogous to the high frequency cut-off used by Ref. [1] to regulate the Fourier inversion integral that arises when solving the diffusion equation. This assigns a radius a to the receptor by truncating the representation of the delta function in the eigenfunction basis, which is the smallest spatial scale in the problem. To proceed analytically we make the simplifying assumption that the

receptor array enjoys a symmetry with respect to Ω such that the sum over μ is independent of ν. For example, this holds for dimensional analogues of a uniform ring about the origin when $\Omega = B^d_R$. Substituting into equation (S.59) and applying the FDT as above, we find a correlation time

$$\tau_N = \frac{1}{\bar{c}k_+ + k_-} + \frac{M\Sigma_{\mathbb{R}}(0, M, \Omega)}{\bar{c}k_+ + k_-}, \tag{S.62}$$

where in this case

$$\Sigma_{\mathbb{R}}(0, M, \Omega) = \frac{k_+(1-\bar{n})}{MD} \sum_{\mu} \sum_{|\alpha|>0}^{\Lambda} \frac{\phi_\alpha(\mathbf{x}_{\boldsymbol{\mu}})\phi_\alpha(\mathbf{x_0})}{||\phi_\alpha||^2} \frac{1}{\lambda_\alpha^2}. \tag{S.63}$$

From this expression the result can be computed for any domain on which the eigenfunctions are known. Although a symmetry assumption is required of the receptor array, setting $M = 1$ also gives a single receptor approximation for which this is trivially satisfied.

Ring of Receptors in 2D

We now extract the result for a ring of receptors in 2D for comparison with that in the main text. We work in polar coordinates on $\Omega = B^2_R$, and consider a uniform ring of receptors at radius $r = b$ and angles $\theta_\mu = \frac{2\pi\mu}{M}$. The Laplacian in this coordinate system has the form

$$\Delta = \frac{1}{r}\frac{\partial}{\partial r}\left(r\frac{\partial}{\partial r}\right) + \frac{1}{r^2}\frac{\partial^2}{\partial \theta^2} \tag{S.64}$$

with eigenfunctions and eigenvalues

$$\phi_{m,l}(r,\theta) = \begin{cases} J_m(\lambda_{m,l} r)\cos(m\theta) \\ J_m(\lambda_{m,l} r)\sin(m\theta) \end{cases} \tag{S.65}$$

$$\lambda^2_{m,l} = \left(\frac{z_{m,l}}{R}\right)^2, \quad m = 0, 1, 2, \ldots \quad l = 0, 1, 2, 3, \ldots \tag{S.66}$$

J_m denotes the Bessel function of the first kind of order m and $z_{m,l}$ are the non-negative zeros of $J'_m(z)$. Thus,

$$\Sigma_{\mathbb{R}}(0, M, b, R) = \frac{k_+(1-\bar{n})}{MD} \sum_{\mu=0}^{M-1} \sum_{m,l}^{\Lambda} \frac{J^2_m(\lambda_{m,l} b)\cos(\frac{2\pi m\mu}{M})}{||\phi_{m,l}||^2} \frac{1}{\lambda^2_{m,l}} \tag{S.67}$$

where we eliminated the sin term by choosing $\mathbf{x_0} = (b, 0)$. The eigenfunction norms can be calculated from the integral formula [2, 11.4.5] as

$$||\phi_{m,l}||^2 = \begin{cases} \pi R^2 J_0^2(z_{0,l}) & m = 0 \\ \dfrac{\pi R^2}{2z^2_{m,l}}\left[z^2_{m,l} - m^2\right] J^2_m(z_{m,l}) & m > 0. \end{cases} \tag{S.68}$$

Further, we can use the properties of roots of unity to show

$$\sum_{\mu=0}^{M-1} \cos(\frac{2\pi m\mu}{M}) = \begin{cases} M & m = kM \\ 0 & m \neq kM, \quad k \in \mathbb{Z} \end{cases}, \tag{S.69}$$

so that

$$\Sigma_{\mathbb{R}}(0,M,b,R) = \frac{k_+(1-\bar{n})}{D\pi}\Big[\sum_{l=1}^{\Lambda}\frac{J_0^2(z_{0,l}\frac{b}{R})}{J_0^2(z_{0,l})}\frac{1}{z_{0,l}^2} + 2\sum_{K,l}^{\Lambda}\frac{J_K^2(z_{K,l}\frac{b}{R})}{J_K^2(z_{K,l})}\frac{1}{z_{K,l}^2 - K^2}\Big], \quad \text{(S.70)}$$

with $K = M, 2M, 3M\ldots$

The sums are over indices such that $\lambda_\alpha < \frac{\pi}{a}$, so the size of the receptors, assumed on the order of nanometres, determines how many terms are included. The first term is independent of the number of receptors, while the second term in this expression (which contains all of the angular dependence) decreases as more receptors are added to the array. However, this noise reduction soon saturates, and the second term becomes insignificant compared to the first. Using the inequality $z_{K,l}^2 > K^2 + 2K$ [3, 15.3.3] in equation (S.70) shows that the second term is $\mathcal{O}(1/M)$ as M becomes large. Furthermore, this inequality places a limit on the highest order of Bessel function in the sum, since for $K > \frac{R\pi}{a}$ all eigenvalues will exceed the cut-off. Since the orders that do appear must be multiples of M, as M increases more terms are removed from the sum. Thus overall we expect fast decay with M, which may be increased up to a maximum $M \sim \frac{\pi b}{a}$ determined by the size of the receptors and the size of the ring. We examine equation (S.70) numerically as a function of M in Supplementary Fig. 5 for fixed R and a and different values of b. We plot the contribution from the first term as a baseline, which shows the approach to this noise floor long before M is near the maximum value.

Since we assume there are many receptors, we therefore neglect the second term and write equation (S.70) as

$$\Sigma_{\mathbb{R}}(0,b,R) \approx \frac{k_+(1-\bar{n})}{D\pi}\sum_{k=1}^{\Lambda}\frac{J_0^2(z_{0,k}\frac{b}{R})}{J_0^2(z_{0,k})}\frac{1}{z_{0,k}^2}. \quad \text{(S.71)}$$

For large k we have $z_{0,k} \approx k\pi + \frac{\pi}{4}$ so that $\Lambda = \left[\frac{R}{a}\right]$. This ensures $\lambda_{0,k} = \frac{z_{0,k}}{R} \leq \frac{\pi}{a}$. The sum converges quickly, so that in the relevant limit $R \gg a$ (and dropping the superfluous subscript) we find

$$\Sigma_{\mathbb{R}}(0,b,R) \approx \frac{k_+(1-\bar{n})}{2\pi D}\sum_{k=1}^{\infty}\frac{J_0^2(z_k\frac{b}{R})}{\frac{1}{2}J_0^2(z_k)}\frac{1}{z_k^2}, \quad \text{(S.72)}$$

This expression, which completely determines the result, is the same as that derived in the main text with closed form given by equation (S.36). This demonstrates that, under our working assumptions, little information is lost when we simplify by homogenisation of the array. This parallels the observation made by Supplementary Ref. [1], that in 3D the noise reduction from adding receptors to an array saturates, so that the array acts as if it is one large receptor.

Supplementary References

[1] Bialek, W. & Setayeshgar, S. Physical limits to biochemical signaling. *Proc. Natl. Acad. Sci. U.S.A.*, **102(29),** 10040-10045 (2005).

[2] Abramowitz, & M., Stegun, I. A. *Handbook Of Mathematical Functions: With Formulas, Graphs, and Mathematical Tables.* (Dover, New York, 1972).

[3] Watson, G. N. *A Treatise on the Theory of Bessel Functions. Reprint of the second (1944) edition.* (Cambridge University Press, Cambridge, 1966).
